# Resistance Patterns, *mcr-4* and *OXA-48* Genes, and Virulence Factors of *Escherichia coli* from Apennine Chamois Living in Sympatry with Domestic Species, Italy

**DOI:** 10.3390/ani12020129

**Published:** 2022-01-06

**Authors:** Camilla Smoglica, Alberto Vergara, Simone Angelucci, Anna Rita Festino, Antonio Antonucci, Lorenzo Moschetti, Muhammad Farooq, Fulvio Marsilio, Cristina Esmeralda Di Francesco

**Affiliations:** 1 Faculty of Veterinary Medicine, University of Teramo, Loc. Piano D’Accio, 64100 Teramo, Italy; avergara@unite.it (A.V.); simone.angelucci@parcomajella.it (S.A.); arfestino@unite.it (A.R.F.); lorenzo.moschetti@studenti.unite.it (L.M.); mfarooq@unite.it (M.F.); fmarsilio@unite.it (F.M.); cedifrancesco@unite.it (C.E.D.F.); 2 Wildlife Research Center, Maiella National Park, Viale del Vivaio, 65023 Caramanico Terme, Italy; antonio.antonucci@parcomajella.it

**Keywords:** wild animals, *E. coli*, carbapenems, colistin, virulence factors, one health

## Abstract

**Simple Summary:**

Antimicrobial resistance is a global threat involving human, animal, and environmental health. Evidence of antibiotic resistance was found in pets, livestock, humans, in uncontaminated environments, or in animals never treated with antibiotics. In order to provide new data, this study was carried out in the protected area of the Maiella National Park (Central Italy) sampling wild and domestic ungulates that share or do not share grazing lands. The analysis was realized by combining the georeferenced data of animals and the microbiological investigations starting from fresh fecal samples. The *Escherichia coli* isolates were tested for antibiotics particularly relevant in human health. Even if the selected molecules are not currently used in veterinary medicine, evidence of resistant bacteria was found in sympatric wild and domestic animals, as well as in non-sympatric domestic animals. The detection of colistin resistance gene *mcr-4* and carbapenems resistance genes *OXA-48* was reported for the first time in wild ungulates in Italy and in Europe. More investigations are necessary, but these preliminary results highlight the importance of continuing studies for the early detection of emerging resistance patterns.

**Abstract:**

The aim of this study was to determine and characterize potential resistance mechanisms against selected Critically Important Antibiotics in *Escherichia coli* isolates collected from wild and domestic ruminants living in the Maiella National Park, in Central Italy. A total of 38 isolates were obtained from red deer, Apennine chamois, cattle, sheep, and goats grazing in lands with different levels of anthropic pressure. Antimicrobial susceptibility was determined by Minimal Inhibitory Concentration testing, showing phenotypic resistance to colistin, meropenem, or ceftazidime in 9 isolates along with one bacterial strain being resistant to three of the tested antibiotics. In addition, the biomolecular assays allowed the amplification of the genes conferring the colistin (*mcr-4*), the carbapenems (*OXA-48*), penicillins and cephalosporins (*TEM*, *SHV*, *CMY-1*, *CMY-2*) resistance. In order to describe the potential pathogenicity of isolates under study, virulence genes related to Shiga toxin-producing (STEC) and enteropathogenic (EPEC) pathovars were identified. This study is the first report of *mcr-4* and *OXA-48* genes in resistant *E. coli* harboring virulence genes in Italian wildlife, with special regard to Apennine chamois and red deer species. The multidisciplinary approach used in this study can improve the early detection of emerging antibiotic resistance determinants in human-animal-environment interfaces by means of wildlife monitoring.

## 1. Introduction

*Escherichia coli (E. coli)* is a regular inhabitant of the microbiota of different hosts, and it is characterized by multiple microbiological roles [1]. Indeed, several pathovars of this bacterium are recognized as a cause of infections in humans and animals and it is possible to classify the bacteria of this species as intestinal non-pathogenic *E. coli,* intestinal pathogenic *E. coli* (IPEC), and extraintestinal pathogenic *E. coli* (ExPEC) [1]. *E. coli* remains one of the most frequent causes of nosocomial and community-acquired bacterial infections in humans, including urinary, and enteric tract, as well as systemic infections, and therapy is complicated by the emergence of antimicrobial resistance [2]. Different studies showed resistant pathogenic *E. coli* to first-line antibiotics, such as first-generation cephalosporins, fluoroquinolones, and trimethoprim-sulfamethoxazole, and to last line β-lactams such as third and fourth generation cephalosporins and carbapenems [1,2]. Direct exposure to antibiotics, contact with animal excretions, or exposure via the food chain have been suggested by other authors as potential transmission pathways of virulent or drug-resistant *E. coli* [3]. The worldwide increase of antimicrobial-resistant *E. coli* represents a challenge to treat infections in humans and animals [4]. Third-generation cephalosporins, carbapenems, and colistin are considered as last resort antibiotics to treat human infections caused by multidrug-resistant gram-negative bacteria and they are included in the list of critically important antimicrobials (CIAs) for human medicine [5]. These antibiotics are of great interest because their application should be targeted for treating the severest human infections in order to preserve their effectiveness. Among them, β-lactams (penicillin, cephalosporins, and carbapenems) are currently used to treat the infections caused by *E. coli* due to their broad-spectrum activity [6]. The cephalosporins are crucial for preventing and treating nosocomial infections but the increased ineffectiveness of third-generation cephalosporins against extended-spectrum beta-lactamases (ESBLs) producing *E. coli* isolates makes the recovery of patients increasingly difficult to achieve [7]. In order to tackle this trend, the carbapenems (such as meropenem and ertapenem) are widely used as alternative treatments of infections caused by multidrug-resistant and ESBL-producing *Enterobacteriaceae* [8].

Indeed, the use of the carbapenems against Gram-negative bacteria is particularly critical in medicine practice considering the emergence of carbapenem-resistant *Enterobacteriaceae* [8,9,10]. Finally, colistin or polymyxin E is a polypeptide antimicrobial agent that may be effective against carbapenem-resistant *E. coli* [11]. Colistin has been used extensively for decades in the treatment and prevention of infectious diseases in veterinary medicine; as a matter of fact, in 2011 polymyxins were the fifth most sold class of antimicrobials for treating food-producing animals in Europe [11]. Resistance to colistin has increasingly been reported among wild and domestic animals [11,12,13,14,15,16,17,18] and a potential horizontal transmission from animals to humans was suggested [19]. In this context, the use of colistin has been regulated in Europe [20] and banned as a growth promoter in China [21]. As a result of this, the sales of polymyxins for veterinary use in Italy have fallen by 97.7% in 2020 compared to 2011 only representing the 0.39% of the annual total sale of antibiotics [22].

Resistant *E. coli* have been widely reported worldwide from humans, livestock, companion animals, and environmental sources as wildlife [3,8,23]. Wild animals are not normally treated with antibiotics but the direct and indirect contact with humans, livestock, domestic animals, or anthropic areas can promote the sharing of commensal or pathogenic bacteria as well as their relative resistance genes [24], highlighting that different ecological niches may have an impact on the dissemination of antimicrobial resistance determinants. Relevant studies were carried out on antimicrobial resistance in wildlife in the last years even if the potential role of wild animals in AMR maintenance is still poorly clear [25].

In this regard, the aim of this study is to investigate the phenotypic and genetic resistance patterns against selected CIAs (cefotaxime, ceftazidime, ertapenem, meropenem, colistin) in *E. coli* isolates recovered from wild and domestic ungulates living in the territories of the Maiella National Park (Central Italy) with different levels of anthropic pressure. In addition, in order to evaluate the potential pathogenicity of bacterial strains under study, specific virulence genes characteristic of different *E. coli* pathovars were attempted.

## 2. Materials and Methods

### 2.1. Study Area

The study area is located within the boundaries of the Maiella National Park (MNP), covering a mountainous area (about 740 km^2^) in the central Apennine Mountains in Italy. The MNP is very close to another two Central Italy Parks: the National Park of Abruzzo, Lazio and Molise (NPALM) and the Gran Sasso and Monti della Laga National Park (GSMLNP). The MNP is the home of several and diversified mammal species relevant at the national and international level and listed in Habitats Directive (92/43/EEC). This is the territory of the rarest Apennine chamois species (*Rupicapra pyrenaica ornata*), Marsican brown bear (*Ursus arctos marsicanus*), and Apennine wolf (*Canis lupus italicus*). In detail, the Apennine chamois lives only in limited areas of Central Italy which are particularly linked to the territories of MNP from where the estimated population of 1500 individuals has originated by the reintroduction activities carried out in the past years. Nevertheless, this species is still facing major threats due to low genetic variation, slow range expansion, and competition with other more widespread wild ungulates (red deer). In addition, in these territories, livestock farming (cattle, sheep. and goats) often relies on the traditional practices, based on small farms and extensive grazing systems, where animals are raised on the mountain pastures.

### 2.2. Sampling Design and Sample Collection

The spatial distribution of wild and domestic animals was determined by georeferenced data and monitoring activities carried out routinely by the technical staff of MNP. These data were provided in order to define the level of grazing land sharing between domestic and wild ruminants. The sympatric animals (Group A) were composed of a sample of 120 goats grazing along with 100 Apennine chamois, and a second group of 300 sheep along with 50 red deer. The segregated animals (Group B) included 70 cattle, 210 goats, 20 red deer, and 100 Apennine chamois were localized in different geographic areas. A total of 33 fecal pools, composed of four single specimens of feces belonging to the same species and population (red deer, Apennine chamois, and livestock) were collected from October to November 2019 (Table 1). In order to improve the recovery of fresh samples and to reduce the risk of soil contamination, the different groups of grazing animals were followed and observed without interfering with the pasture, collecting only fresh feces related to the animals. The samples were stored at 4° C and analyzed within 24 h of the collection at the laboratory of the Faculty of Veterinary Medicine of the University of Teramo.

### 2.3. Bacteria and Antibiotic Susceptibility Test

Bacterial colonies were obtained by a preliminary non-selective enrichment of fecal samples in buffered peptone water (24 h at 37 °C), followed by subculture on MacConkey agar (Liofilchem, Italy) at 37 °C for 18–24 h. From each plate, 1 or 2 representative colonies, morphologically referred to as *E. coli* were selected. The species identification was performed by means of the Vitek 2 system (Biomerieux, Marcy-l’Étoile, France), and the antimicrobial susceptibility tests for cefotaxime (CTX), ceftazidime (CAZ), ertapenem (ETP), meropenem (MRP), and colistin (CS) were carried out by means of MIC Test strip (Liofilchem, Roseto degli Abruzzi, Italy). Considering that the Veterinary Committee on Antimicrobial Susceptibility Testing (VetCAST) breakpoints are not currently defined for CIAs under study, the European Committee on Antimicrobial Susceptibility Testing (EUCAST) breakpoints relevant for human health were applied [26]. In addition, the wild-type strains were characterized using the epidemiological cut-off values (ECOFF) as defined by the European Committee on Antimicrobial Susceptibility testing (CTX: ≤0.25 μg/mL, CAZ: ≤0.5 μg/mL, ETP: ≤0.03 μg/mL, MRP: ≤0.06 μg/mL and CS ≤ 2 μg/mL) [27].

### 2.4. Detection of Antibiotic Resistance and Virulence Factors Genes

The genes coding for β-lactamases and Extended-spectrum β-lactamases (ESBLs) (*bla*TEM, *bla*SHV, *bla*CTX-M), AmpC type β-lactamases (AmpCs) (*bla*CMY-1, *bla*CMY-2), carbapenems (*IMP*, *OXA-48*, *NDM*, *KPC*) and colistin (*mcr-1*, *mcr-2*, *mcr-3*, *mcr-4*, *mcr-5*), along with the virulence factors *stx1*, *stx2*, *escV*, *eaeA*, *astA*, *hlyA* were screened by PCR (Table 2). The virulence genes were selected based on the pathovars yet reported in wildlife in Central Italy [28]. In order to confirm the specificity of PCR results, the amplicons were purified by means of the QIAquick Gel Extraction Kit (Qiagen, Germany), submitted to the Sanger sequencing, and compared with analogous sequences included in the EMBL database using the CHROMAS software, FASTA (http://www.ebi.ac.uk/fasta33 (accessed on 30 September 2021)), Clustal Omega (http://www.ebi.ac.uk/Tools/msa/clustalo (accessed on 30 September 2021)), and Basic Local Alignment Search Tool (BLAST). The sequences obtained were submitted to the Genbank database under the accession number from OL872167 to OL872179 and OL840852.

## 3. Results

In all the fecal pools one or more *E. coli* strains were isolated. In detail, 14 isolates were obtained from the samples belonging to the group of sympatric animals (4 red deer; 4 Apennine chamois; 3 sheep; 3 goats;) and 24 from the group of non-sympatric animals (10 Apennine chamois; 7 cattle; 6 goats; 1 red deer). The MIC values of CTX, CAZ, ETP, MRP, and CS were determined for *E*. *coli* recovered from the two groups of animals (Table 3). Out of 38 isolates, 10 *E. coli* strains (26.31%) exhibited resistance to CS, with 6/10 (60%) of the isolates belonging to sympatric animals and 4/10 (40%) from non-sympatric animals. In detail, phenotypic resistant isolates were detected in four wild sympatric, two domestic sympatric, and four domestic non-sympatric animals. Only one *E. coli* showed multiple resistance to CS, CAZ, and MRP. This isolate was detected from Apennine chamois belonging to the sympatric animal group. Based on the ECOFF values, all sensitive isolates were considered not wild type for MRP and ETP.

Among the β lactamase resistance genes investigated by PCR test, *bla*CMY-2 was detected in 14/38 (36.8%) isolates, *bla*TEM in 8/38 (21%), and the gene *bla*CMY-1 in 8/38 (21%) isolates. Finally, the *bla*SHVgene was amplified in only one isolate deriving from sympatric Apennine chamois, and the sequence analysis allowed to identify the variant blaSHV-2 (Genbank Accession number: OL872171). In total, the β lactamase resistance genes were amplified from 10 isolates recovered from the sympatric group and eight isolates from non-sympatric animals. As reported in Table 3, the β lactamase resistance genes were amplified in eight domestic non-sympatric animals and in six wild and four domestic sympatric animals. The sequence analysis of *bla*TEM amplicons returned results consistent for *bla*TEM-135, *bla*TEM-57, and *bla*TEM-1 genes (Genbank accession numbers: OL872168, OL872169, OL872170).

All the *E. coli* under study were negative for carbapenems resistance genes except for 1 isolate resistant to MRP in which the *bla*OXA-48 gene was detected.

Finally, the only colistin resistance gene *mcr-4* was amplified in 9/38 (23.7%) isolates, involving five isolates from sympatric and four from not sympatric ruminants (Table 3). In detail, the *mcr-4* gene was identified in three isolates from wild and two isolates from domestic sympatric animals, and in four isolates from non-sympatric domestic animals.

Furthermore, 35 out of 38 isolates (92.1%) were positive for at least one of the virulence genes investigated. In detail, the most representative gene was *astA* (30/38; 78.9%), followed by *hlyA* (20/38; 52.6%), *stx1* (12/38; 31.57%), and *stx2* (11/38; 28.9%) genes. On the contrary, the *eaeA* and *escV* genes were amplified only in 1 *E. coli* isolate. Table 3 shows the co-occurrence of genes detected in the samples under study.

In agreement with the classification previously described [39], 16 isolates (42.1%), showing positivity for *Stx* family subgroups (stx1 and/or stx2 genes), were recognized as Shiga toxin-producing *E. coli* (STEC). STEC isolates were identified in six isolates from wild non-sympatric animals, in three isolates from wild sympatric animals, in five isolates from domestic non-sympatric animals, and in two isolates from domestic sympatric animals. Finally, a single isolate from non-sympatric Apennine chamois showed *escV* gene along with *eaeA* gene, encoding for virulence determinants in enteropathogenic (EPEC) pathovar.

## 4. Discussion

Antimicrobial resistance (AMR) in wildlife is a research topic that has attracted particular interest in recent years [17]. Indeed, several authors have previously tested *E. coli* strains isolated from free-ranging terrestrial wild mammals and, more recently, the studies are focused on the AMR against the last resource antibiotics having particular impact on Public Health [24,25].

The phenotypic colistin resistance and related *mcr* genes were reported in wild mammals including fallow deer in Europe [17], Barbary macaques in Africa [13] and Père David’s deer in Asia [14]. In Italy, phenotypic resistance to colistin without evidence of resistance genes was reported in isolates from hunted wild boars in Emilia Romagna [18], while phenotypic resistance along with *mcr-1* and *mcr-2* genes were recently detected in hunted wild boars in Tuscany [16]. In this study, the phenotypic resistance to colistin was reported in isolates from sympatric domestic and wild ungulates, while in the group of non-sympatric animals it was only detected in livestock. In addition to the phenotypic resistance, it was reported the occurrence of *mcr-4* gene. In Italy, *mcr-4* first characterization was in *Salmonella enterica* serovar Typhimurium in a pig [12], afterward, *mcr-4* was also reported in *Salmonella enterica* serovar Typhimurium from human patients with gastroenteritis [40], in *E. coli* from various stages of the broiler production pyramid (breeder, manure and soil), [15] and in *Enterobacter cloacae* from a hospitalized elderly woman [41]. Based on the above research, our study represents the first report of *mcr-4* occurrence in wildlife with particular regard to red deer and Apennine chamois species.

The phenotypic resistance to cephalosporins has been investigated worldwide and the host taxa that carry cephalosporinases in Europe include birds, mammals, reptiles, fish, and mollusks [8]. Considering wild mammals, the *bla*SHV has been reported in Gram-negative bacteria with reduced susceptibility to third-generation cephalosporins from wild boars, European hedgehogs, beech marten, and European badgers in Spain and Italy [42,43,44]. The *blaTEM* gene was reported in resistant *E. coli* from wild boars in Portugal, Poland, and Spain and it was considered by some authors the most frequent gene conferring resistance to β-lactams [43,45,46]. Otherwise, in Italy, a low prevalence of *bla*TEM was reported in resistant isolates from wild boars collected in Emilia Romagna and Lombardy regions [18,44]. Previously, the *bla*CMY-1 was mainly detected in avian wildlife [42] and with a low prevalence in resistant *E. coli* from wild boars in Spain [43]. Regarding *bla*CMY-2, it was earlier reported in hedgehogs, roe deer, and American minks in Spain [42], while in Italy the *bla*CMY-2 gene was found in wild boars, badgers, wolves, foxes, and mouflons [18,28,44]. The detection of *bla*SHV, *bla*TEM, *bla*CMY-1, and *bla*CMY-2 in this study is in line with the previous reports and it allows the addition of red deer and Apennine chamois to the list of European wild species showing this genetic resistance profile [18,28,42,43,44]. Similarly to what was observed for colistin, these genes were found in both wild and domestic animals of group A and in domestic animals of group B. Indeed, the phenotypic resistance to third-generation cephalosporins was reported in only one isolate of Apennine chamois from group A, resulting in phenotypic resistant even to colistin and meropenem.

To the best of our knowledge, carbapenems resistant bacteria were reported in wildlife (from Germany, France, and south-east Australia) especially in wild birds [46]. Regarding wild mammals, the phenotypic carbapenems resistance along with the *bla*OXA-48 gene have been previously reported in bacteria from wild boars in Algeria [47] and in hedgehog and mustelids in Spain [42]. In this view, this is the first report of *bla*OXA-48 in ungulates in Italy and in Europe.

Considering that all the isolates under study were characterized as not wild type for ERT and MRP, in accordance with ECOFFs values, the monitoring of emerging resistance patterns in human/animal interfaces should be improved.

The multi-disciplinary approach applied in this study allows the realization of a snapshot of the environmental contamination in the examined territory. Indeed, the analysis was carried out by collecting not just the opportunistic samples from georeferenced free-ranging animals, highlighting the occurrence of resistant *E. coli* in domestic and wild animals of group A. and only in domestic species of group B. The lack of resistant isolates in wild animals of non-sympatric group B provides additional information about the potential role of human-related activities in the dynamics of AMR spread, suggesting that the interactions with livestock could enhance the share of resistant bacteria with wildlife.

It is relevant to note the presence of virulence genes in isolates from A and B groups, including both wild and domestic animals. These genes provide virulence attributes that increase bacterial adaptability to act as pathogenic agents [17]. In detail, domestic ruminants are considered natural reservoirs of STEC pathovar [48], being clinically tolerant due to the lack of *Stx* receptors in the intestinal tract and a lower receptivity in the kidney and brain [49]. A similar pathotype was previously reported in Italy from samples opportunistically collected from wild boars, badgers, wolves, foxes, mouflon in Tuscany and from red deer during the culling plan in the Stelvio National Park [28,50], suggesting potential interspecies transmission of isolates as a result of wildlife-livestock overlapping [50]. However, the STEC isolates reported in this study were mainly detected in wild non-sympatric animals raising questions about the real pathways of virulent bacteria spreading [51]. As previously suggested by other authors, the multi-virulent profiles observed in wild animals of MNP may be associated with the adaptation to the hosts rather than to the share of grazing land with livestock [52]. However, the pathogenic risk for endangered wild species or humans cannot be completely ruled out considering that some strains harboring virulence factors are also resistant to the antibiotics relevant for Public Health. Indeed, the fecal contamination of soil and water from wild or domestic ruminants may be a source of exposure for humans and animals to STEC variants [48,50,51].

In the future, the analysis of the whole genome of these isolates could improve our knowledge of the relationship among bacteria, environmental sources, and animals investigated. Doubtlessly, the occurrence of CIAs resistant *E. coli*, positive for several virulence genes, confirms the need to investigate the environmental impact of human activities, with particular regard to the food-producing livestock industry, and their role as potential sources of AMR [24,53].

## 5. Conclusions

The phenotypic and genotypic resistance observed in this study were recovered in domestic and wild animals that share the grazing land, and in non-sympatric domestic animals. In wild animals with limited contact with human activities, the resistance patterns under study were not detected. Based on our results, the investigated wildlife may be considered an indicator of emerging antibiotic resistance patterns considering the different levels of human/animal interactions observed in the study area. Additional studies comparing human- and animal-origin strains should be encouraged in order to assess the importance of human/animal interactions in the transmission of antibiotic resistance to organisms.

## Figures and Tables

**Table 1:** Number of fecal pools obtained from each species of sympatric (A) and non-sympatric (B) groups.

Groups	Animals	Number of Fecal Pools
A	Goat (n = 120)	3
	Apennine chamois (n = 100)	2
	Sheep (n = 300)	3
	Red deer (n = 50)	4
B	Cattle (n = 70)	5
	Goat (n = 210)	8
	Red deer (n = 20)	3
	Apennine chamois (n = 100)	5

**Table 2:** Details of oligonucleotides used for the detection of antimicrobial resistance and virulence factors genes.

Primer	Sequence 5′-3′	Size (bp)	Annealing	Ref.
*Mcr-1* Fw	AGTCCGTTTGTTCTTGTGGC	320	56 °C	[29]
*Mcr-1* Rev	AGATCCTTGGTCTCGGCTTG			
*Mcr-2* Fw	CAAGTGTGTTGGTCGCAGTT	715		
*Mcr-2* Rev	TCTAGCCCGACAAGCATACC			
*Mcr-5* Fw	ATGCGGTTGTCTGCATTTATC	207	56 °C	[30]
*Mcr-5* Rev	TCATTGTGGTTGTCCTTTTCTG			
*Mcr-4* Fw	ATTGGGATAGTCGCCTTTTT	487	50 °C	[12]
*Mcr-4* Rev	TTACAGCCAGAATCATTATCA			
*Mcr-3* Fw	AAATAAAAATTGTTCCGCTTATG	542	50 °C	[31]
*Mcr-3* Rev	AATGGAGATCCCCGTTTTT			
*bla*TEM F	CCGTGTCGCCCTTATTCCC	780	51 °C	[32]
*bla*TEM R	GCCTGACTCCCCGTCGTGT			
*IMP*-F	GGAATAGAGTGGCTTAAYTCTC	232	56 °C	[33]
*IMP*-R	GGTTTAAYAAAACAACCACC			
*OXA-48* F	GCGTGGTTAAGGATGAACAC	438		
*OXA-48* R	CATCAAGTTCAACCCAACCG			
*NDM*-F	GGTTTGGCGATCTGGTTTTC	621		
*NDM*-R	CGGAATGGCTCATCACGATC			
*KPC*-F	CGTCTAGTTCTGCTGTCTTG	790		
*KPC*-R	CTTGTCATCCTTGTTAGGCG			
*bla*CTX-M F	ATGTGCAGYACCAGTAARGTKATGGC	593	60 °C	[34]
*bla*CTX-M R	TGGGTRAARTARGTSACCAGAAYCAGCGG			
*bla*CMY-2 F	GCACTTAGCCACCTATACGGCAG	758		
*bla*CMY-2 F	GCTTTTCAAGAATGCGCCAGG			
*bla*SHV F	TTATCTCCCTGTTAGCCACC	797	60 °C	[35]
*bla*SHV R	GATTTGCTGATTTCGCTCGG			
*bla*CMY-1 F	ATGCAACAACGACAATCC	1085	58 °C	[36]
*bla*CMY-1 R	TTGGCCAGCATGACGATG			
*escV* F	ATTCTGGCTCTCTTCTTCTTTATGGCTG	544	62 °C	[37]
*escV* R	CGTCCCCTTTTACAAACTTCATCGC			
*astA* F	TGCCATCAACACAGTATATCCG	102		
*astA* R	ACGGCTTTGTAGTCCTTCCAT			
*eaeA* F	GACCCGGCACAAGCATAAGC	384	65 °C	[38]
*eaeA* R	CCACCTGCAGCAACAAGAGG			
*stx1* F	ATAAATCGCCATTCGTTGACTAC	180		
*stx1* R	AGAACGCCCACTGAGATCATC			
*stx2* F	GGCACTGTCTGAAACTGCTCC	255		
*stx2* R	TCGCCAGTTATCTGACATTCTG			
*hlyA* F	GCATCATCAAGCGTACGTTCC	534		
*hlyA* R	AATGAGCCAAGCTGGTTAAGCT			

**Table 3:** Details of animal source, Minimum Inhibitory Concentration (MIC) values, resistance and virulence genes among 38 *E. coli* isolates from two groups of wild and domestic animals.

Group *	Source	MIC (μg/mL) **					Genes	
		CTX	CAZ	ETP	MRP	CS	Resistance	Virulence
A Wild (n = 8)	Red deer	0.25	0.12	0.12	0.25	2	*bla*TEM, *bla*CMY-2	*astA*
	Red deer	0.25	0.12	0.12	0.25	2	*bla*TEM, *bla*CMY-2	
	Red deer	0.25	0.12	0.12	0.25	**3**	*bla*CMY-2, *mcr-4*	*hlyA*
	Red deer	0.25	0.12	0.12	0.25	**3**	*bla*CMY-2, *mcr-4*	*astA*, *stx2*, *hlyA*
	Chamois	0.25	0.12	0.12	0.25	2	*bla*SHV	
	Chamois	0.5	**64**	0.12	**4**	**3**	*bla*TEM, *bla*CMY-1, *mcr-4*, *bla*OXA-48	*stx1*, *hlyA*
	Chamois	0.25	0.25	0.12	0.25	1.5		*hlyA*
	Chamois	0.25	0.25	0.12	0.25	**3**		*astA*, *stx1*, *stx2*, *hlyA*
A Domestic (n = 6)	Sheep	0.25	0.12	0.12	0.25	2	*bla*CMY-2	*astA*
	Sheep	0.25	0.12	0.12	0.25	2	*bla*CMY-1, *bla*CMY-2	*astA*
	Sheep	0.25	0.12	0.12	0.25	**3**	*bla*TEM, *bla*CMY-2, *mcr-4*	*astA*
	Goat	0.25	0.12	0.12	0.25	1.5	*bla*CMY-2	*astA*, *stx1*, *stx2*
	Goat	0.25	0.12	0.12	0.25	2		*astA*
	Goat	0.25	0.12	0.12	0.25	**3**	*mcr-4*	*astA*, *hlyA*, *stx1*
B Wild (n = 11)	Red deer	0.25	0.12	0.12	0.25	2		*astA*
	Chamois	0.25	0.25	0.12	0.25	1.5		*astA*, *stx1*, *hlyA*
	Chamois	0.25	0.25	0.12	0.25	1.5		*astA*, *stx1*, *hlyA*
	Chamois	0.25	0.12	0.12	0.25	1.5		*astA*, *stx2*, *hlyA*
	Chamois	0.25	0.12	0.12	0.25	2		*escV*, *eaeA*, *hlyA*
	Chamois	0.25	0.12	0.12	0.25	1.5		
	Chamois	0.25	0.12	0.12	0.25	1.5		*astA*, *stx1*, *stx2*, *hlyA*
	Chamois	0.25	0.25	0.12	0.25	2		*astA*, *stx1*, *stx2*, *hlyA*
	Chamois	0.25	0.12	0.12	0.25	2		*astA, hlyA*
	Chamois	0.25	0.25	0.12	0.25	2		*astA*, *stx1*, *stx2*, *hlyA*
	Chamois	0.25	0.12	0.12	0.25	2		*astA*
B Domestic (n = 13)	Goat	0.25	0.25	0.12	0.25	1.5		*astA*
	Goat	0.25	0.25	0.12	0.25	1.5		*astA*
	Goat	0.25	0.12	0.12	0.25	2	*bla*CMY-1, *bla*CMY-2	*astA, hlyA*
	Goat	0.25	0.12	0.12	0.25	1.5		*astA*
	Goat	0.25	0.12	0.12	0.25	1.5	*bla*CMY-1, *bla*CMY-2	*astA*, *stx2*, *hlyA*
	Goat	0.25	0.12	0.12	0.25	1.5		*astA*, *stx1*, *hlyA*
	Cattle	0.25	0.12	0.12	0.25	**256**	*bla*TEM, *mcr-4*	*astA*
	Cattle	0.25	0.25	0.12	0.25	1.5	blaTEM	*astA*
	Cattle	0.25	0.12	0.12	0.25	2	*bla*TEM, *bla*CMY-1, *bla*CMY-2, *mcr-4*	*astA*
	Cattle	0.25	0.12	0.12	0.25	0.75	*bla*TEM, *bla*CMY-1, *bla*CMY-2	*astA*, *stx1*, *stx2*, *hlyA*
	Cattle	0.25	0.12	0.12	0.25	**3**	*bla*CMY-1, *bla*CMY-2, *mcr-4*	*astA*, *stx1*, *stx2*, *hlyA*
	Cattle	0.25	0.12	0.12	0.25	**3**	*bla*CMY-1, *bla*CMY-2	*stx2*, *hlyA*
	Cattle	0.25	0.12	0.12	0.25	**3**	*mcr-4*	*astA*

* A: sympatric animals; B: non-sympatric animals; ** CTX: cefotaxime; CAZ: ceftazidime; MRP: meropenem; ETP: ertapenem; CS: colistin. The MIC values above the EUCAST breakpoints are highlighted in bold.

## Data Availability

The data presented in this study are contained within the article and further information will be made available upon reasonable request to the corresponding author.
